# Genomic profile of eGFP-expressing canine distemper virus that undergoes serial plaque-to-plaque transfers

**DOI:** 10.3389/fcimb.2022.1006273

**Published:** 2022-09-23

**Authors:** Jiahui Lin, Yujia Jiang, Hui Zhang, Feng Zhang, Youming Zhang, Bo Ni, Fuxiao Liu

**Affiliations:** ^1^ College of Veterinary Medicine, Qingdao Agricultural University, Qingdao, China; ^2^ Surveillance Laboratory of Livestock Diseases, China Animal Health and Epidemiology Center, Qingdao, China; ^3^ State Key Laboratory of Microbial Technology, Shandong University, Qingdao, China

**Keywords:** canine distemper virus, eGFP, next-generation sequencing, plaque-to-plaque transfer, Muller’s ratchet

## Abstract

Canine distemper virus (CDV) is classified into the genus *Morbillivirus* in the family *Paramyxoviridae*. This virus has a single-stranded genomic RNA with negative polarity. The wild-type CDV genome is generally composed of 15 690 nucleotides. We previously rescued an enhanced green fluorescence protein (eGFP)-tagged recombinant CDV (rCDV-eGFP) using reverse genetics. In this study, the rCDV-eGFP at passage-7 was subjected to 38 serial plaque-to-plaque transfers (or bottleneck passages) and two extra common passages in cells. In theory, the effect of Muller’s ratchet may fix deleterious mutations in a single viral population after consecutive plaque-to-plaque transfers. In order to uncover a mutated landscape of the rCDV-eGFP under the circumstances of bottleneck passages, the passage-47 progeny was collected for the in-depth analysis *via* next-generation sequencing. The result revealed a total of nine single-nucleotide mutations (SNMs) in the viral antigenome. Out of them, SNMs at nt 1832, 5022, 5536, 5580, 5746, 6913 and 8803 were identified as total single-nucleotide substitution, *i.e.*, 100% of mutation frequency. The result suggested no notable formation of viral quasispecies in the rCDV-eGFP population after consecutive plaque-to-plaque transfers.

## Introduction

Canine distemper virus (CDV), now renamed canine morbillivirus, is assigned taxonomically to the family *Paramyxoviridae*, the genus *Morbillivirus*. This virus, as the etiological agent of canine distemper, is associated with multiple cell tropisms, causing a systemic infection, including respiratory, digestive, urinary, lymphatic, cutaneous, skeletal, and central nervous system diseases (Lempp et al., 2014; Rendon-Marin et al., 2019). CDV virion is a pleomorphic particle, containing a single-stranded RNA with negative polarity. The wild-type CDV genome, composed of 15,690 nucleotides (nt), has six transcriptional units, independently coding for six structural proteins, namely nucleocapsid (N) protein, phosphoprotein (P), matrix (M) protein, fusion (F) protein, hemagglutinin (H) and large (L) protein (Duque-Valencia et al., 2019).

The CDV genome is fully encapsidated by numerous N proteins to form a helical nucleocapsid. The encapsidation process obeys the “rule of six” of paramyxovirus (Kolakofsky et al., 2005). The nucleocapsid is additionally combined with P and L proteins, forming a ribonucleoprotein complex. The L protein is also known as RNA-dependent RNA polymerase (RdRp), the largest but the least abundant of the virus proteins. Morbilliviral RdRp is assumed to carry all activities necessary for RNA transcription and genomic (antigenomic) replication (Barrett et al., 2006). Due to its characteristics of low fidelity for genomic and antigenomic replications, random mutations would unavoidably arise during morbilliviral propagation (Liu et al., 2016).

The theory, that is, small asexual populations of organisms will tend to accumulate irreversible deleterious mutations unless genetic lesions are repaired by sex or recombination, was first proposed by Muller (Muller, 1932; Muller, 1964), and afterwards named “Muller’s ratchet” (Felsenstein, 1974; Haigh, 1978). If a population with asexual genome undergoes a series of bottleneck events, such as consecutive plaque-to-plaque (genetic bottleneck) transfers of a given virus, the effects of Muller’s ratchet would be accentuated (Jaramillo et al., 2013). Plaque-to-plaque transfers are involved in isolation of a virus-induced plaque for harvesting a single viral progeny, which will be serially diluted with medium and subsequently seeded on cell monolayers for generating next-generation plaques, from which one typical plaque will be picked out for the next round of plaque-to-plaque passaging (Manrubia and Lázaro, 2006).

Owing to the effect of Muller’s ratchet, serial plaque-to-plaque transfers may fix deleterious mutations in a single population, and then result in the gradual loss of viral fitness in cells, or even induce a viral error catastrophe (Chao, 1990; Duarte et al., 1992; Clarke et al., 1993; Escarmís et al., 1996; Yuste et al., 1999). For example, ten clones of human immunodeficiency virus type 1 were subjected to consecutive plaque-to-plaque transfers, resultantly showing that two clones stopped forming plaques at passage-7 (P7), two others stopped at P13, and only four of the remaining six clones yielded infectious viruses (Yuste et al., 1999). Another example was the vesicular stomatitis virus: following only 20 plaque-to-plaque transfers, most viral clones were proven to undergo significant fitness losses in cells, despite others exhibiting no dramatic fitness alteration (Duarte et al., 1992).

Enhanced green fluorescent protein (eGFP) has been widely used as a reporter to rescue recombinant viruses *via* reverse genetics technique. We previously rescued an eGFP-tagged recombinant CDV (rCDV-eGFP) from a recombinant CDV cDNA clone. This cDNA clone, genetically derived from that of the CDV 5804P strain (Genbank access No.: AY386316), contained an eGFP transcriptional unit, composed of one open reading frame (ORF) and two untranslated sequences. The rCDV-eGFP had been subjected to 47 common passages *in vitro* for next-generation sequencing (NGS) analysis. The NGS technique has revolutionized the depth of virological research, due to its ability to map an exceedingly detailed landscape of viral population structure (Seifert and Beerenwinkel, 2016). Therefore, our previous study by means of the NGS unraveled a complete profile of single-nucleotide mutations (SNMs) in the antigenome of rCDV-eGFP at P47 (Liu et al., 2021b).

In the present study, the P7 rCDV-eGFP was subjected to 38 consecutive plaque-to-plaque transfers and two extra common passages *in vitro*. The P47 progeny was collected for NGS analysis, which uncovered a mutated landscape of viral antigenome under the circumstances of bottleneck passages. Such a mutated landscape was demonstrated to be totally different from that of the same virus previously undergoing 47 common passages *in vitro*.

## Materials and methods

### Cell and virus

Vero-Dog-SLAM (VDS) cell line was cultured at 37°C with 5% CO_2_ in Dulbecco’s modified Eagle’s medium (DMEM) supplemented with 10% fetal bovine serum (VivaCell, Shanghai, China), and containing penicillin (100 U/mL), streptomycin (100 µg/mL), amphotericin B (0.25 µg/mL) and G418 (500 µg/mL). The rCDV-eGFP was rescued previously (Liu et al., 2021b), and the P7 progeny was used here.

### Serial plaque-to-plaque transfers

**Figure 1:**
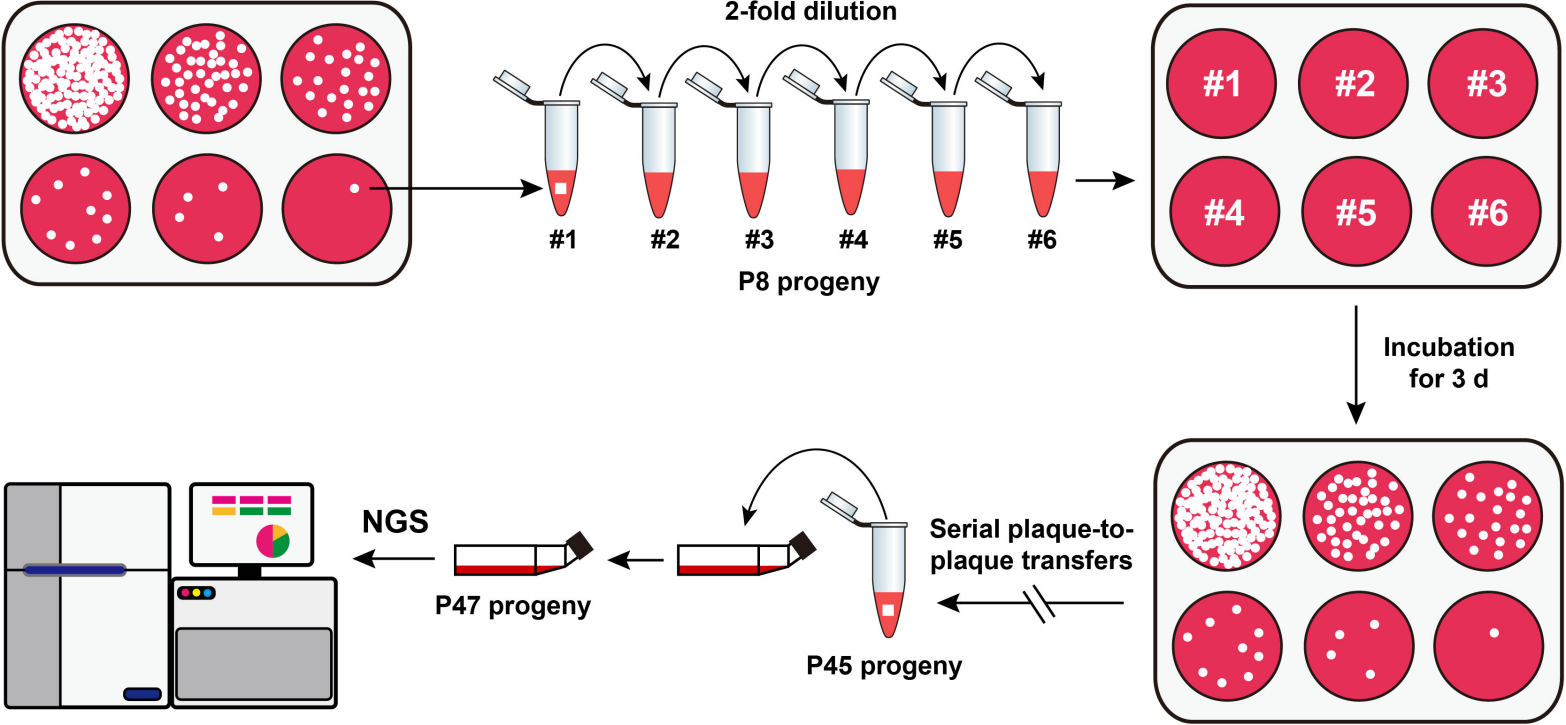
Schematic representation of serial viral passaging for NGS analysis. The P7 rCDV-eGFP is subjected to 38 consecutive plaque-to-plaque transfers (P8 to P45) and two extra common passages (P46 and P47) *in vitro*. The P47 viral stock is harvested for NGS analysis.

The rCDV-eGFP was subjected to repeated plaque-to-plaque transfers *in vitro*, as schematically shown in **Figure 1**. In brief, confluent VDS cell monolayers in a 6-well plate were inoculated with the P7 rCDV-eGFP stock that was serially 2-fold diluted in DMEM. The culture supernatants were separately removed at 2 h post inoculation (hpi), immediately followed by overlaying the cell monolayers with pre-warmed DMEM that contained 1% low-melting-point agarose. The 6-well plate was incubated at 37°C with 5% CO_2_. Viral plaques were observed using a fluorescence microscope at 72 hpi, and then a single green plaque, irrespective of its brightness and size, was randomly picked out from a cell monolayer for resuspension in a DMEM-containing tube. The re-supernatant was subjected to one freeze-and-thaw cycle for the next round of plaque-to-plaque transfer. Genetic bottleneck passages of rCDV-eGFP were carried out by 38 serial plaque-to-plaque transfers (P8 to P45) in VDS cells.

### NGS and data analysis of rCDV-eGFP at P47

For enhancing the viral titer, the P45 plaque-transferred progeny underwent extra two common passages in T25 flasks. The P47 culture supernatant was harvested after one freeze-and-thaw cycle for extracting total RNAs using the Viral RNA/DNA Extraction Kit (Takara, Dalian, China), according to the manufacturer’s instruction. The RNA sample was reverse transcribed by random hexamers using the HiScript^®^ 1st Strand cDNA Synthesis Kit (Vazyme, Nanjing, China), according to the manufacturer’s instruction. The Illumina sequencing was performed to construct a library, followed by the further analysis of NGS data, which included a series of steps, such as preprocessing/quality control, filtering sequences, assembly, taxonomic identification, and validation/analysis, as described in previous reports (Nurk et al., 2013; Cantalupo and Pipas, 2019; Liu et al., 2021a).

In brief, raw reads were filtered *via* the fastp to remove sequencing adapters and low-quality reads, including those reads scored less than Q20. The BBMap program was used to subtract ribosomal RNAs and host reads through read-mapping*. De novo* assembly of the viral antigenome was carried out by means of the SPAdes v3.14.1. The extracted assembled scaffolds limited the minimum contig length to 100 bases, with the best BLAST hits to the NCBI nucleotide database. High-quality filtered reads were mapped against the full-length sequence of rCDV-eGFP antigenome by Burrows-Wheeler Aligner v0.7.17, which also generated a BAM file to calculate the mapping depth and coverage. SNMs were identified using an integrated software package, Snippy v4.4.5, including both substitutions and insertions/deletions. The available SNM results would be selected, if mapping quality was ≥ 60 and depth was ≥ 10.

## Results and discussion

### The rCDV-eGFP can be stably passaged in plaque-to-plaque pattern

**Figure 2:**
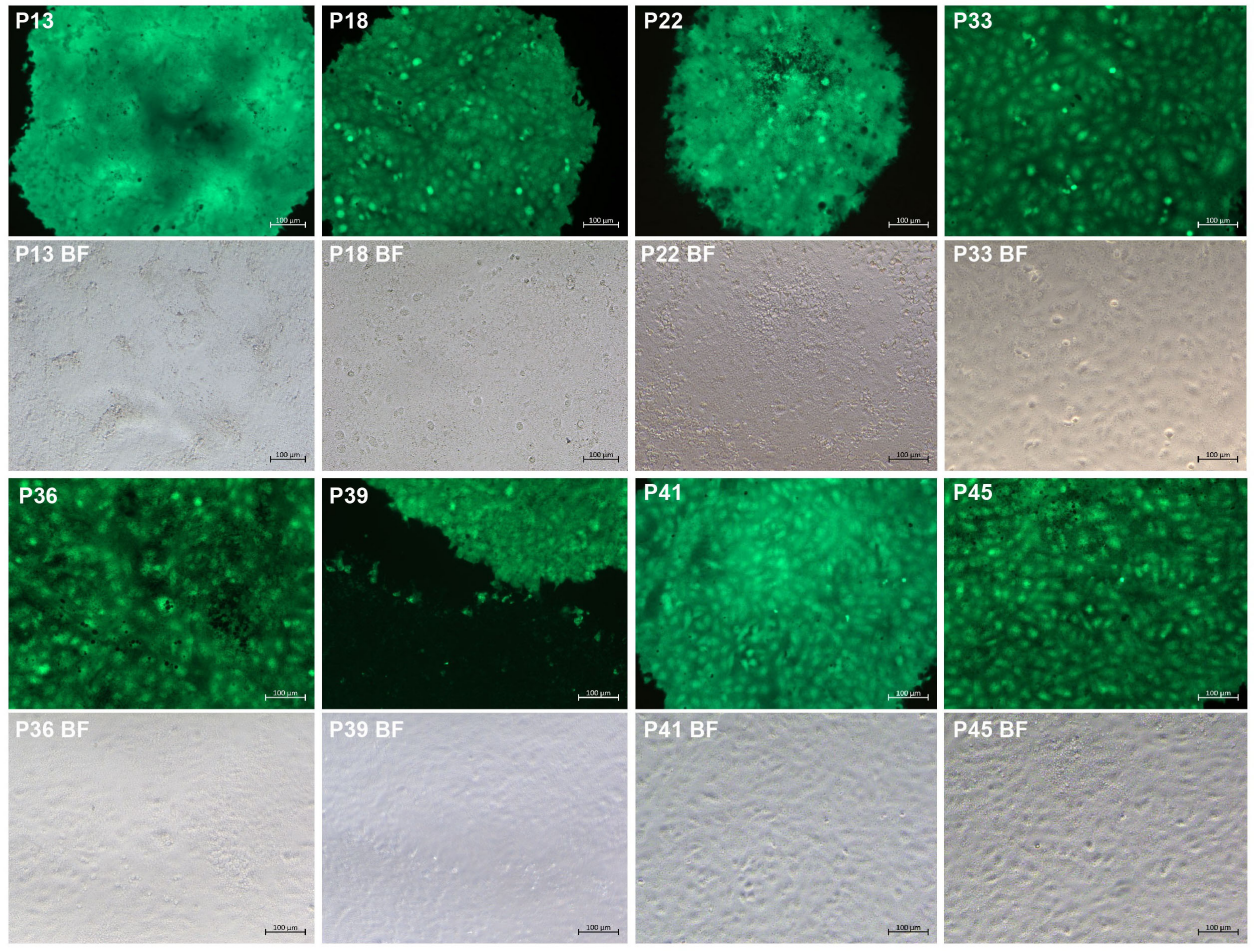
Green plaque formation during serial plaque-to-plaque transfers in VDS cell monolayers. BF, bright field.

The eGFP is widely used as a reporter to rescue recombinant viruses. Its fluorescence characteristics make possible real-time tracking of virus infection. The P7 rCDV-eGFP was able to induce bright green fluorescence on a VDS cell monolayer (Liu et al., 2021b). Here, it underwent a total of 38 plaque-to-plaque transfers (P8 to P45). Different CDV strains may induce differential plaque sizes (Cosby et al., 1981). In the present study, green plaques were always visible on VDS cell monolayers by observation using the fluorescence microscope, while the plaque sizes were not completely consistent with one another (**Figure 2**). In addition, fluorescent plaques simultaneously contained bright and dim phenotypes on a cell monolayer at times. In any case, one fluorescent plaque was randomly picked out from a well for the next round of plaque assay. Interestingly, even if one dim green plaque was picked out for passaging, the next-generation phenotype still contained bright plaques. The plaque-transferred progeny was demonstrated to have an extremely low titer at P45 (data not shown). Therefore, in order to harvest a high-titer stock after plaque-to-plaque transfers for NGS analysis, the P45 progeny was subjected to two extra common passages in T25 flasks.

### NGS shows analyzable sequencing depths

**Figure 3:**
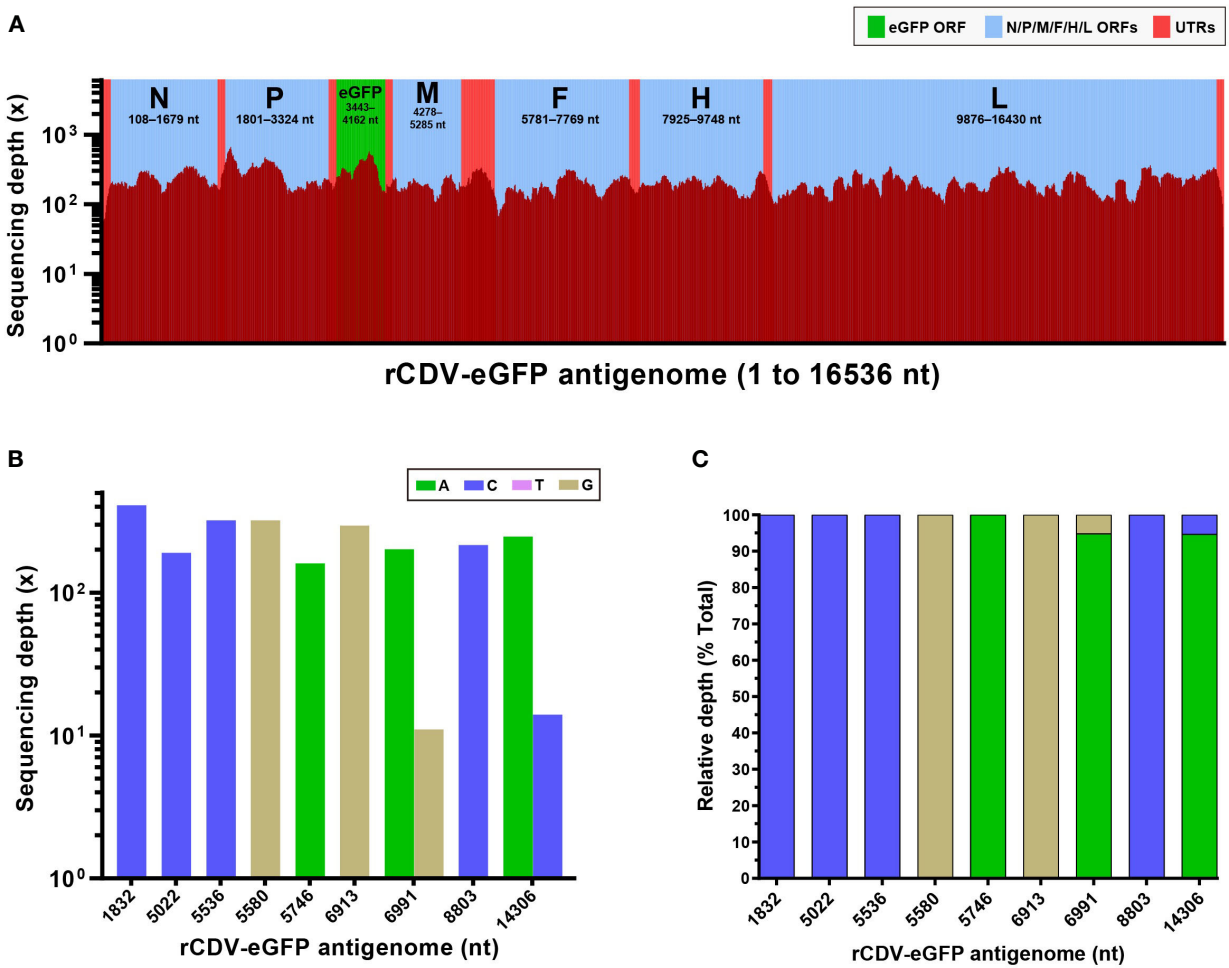
NGS analysis of rCDV-eGFP population at P47. Sequencing depth and coverage of NGS for the full-length rCDV-eGFP antigenome at P47 **(A)**. All elements proportionally match their actual lengths within the viral antigenome. ORF, open reading frame; UTR, untranslated region. Absolute **(B)** and relative **(C)** sequencing depths of SNMs in the rCDV-eGFP antigenome at P47. SNMs at nt 1832, 5022, 5536, 5580, 5746, 6913 and 8803 are recognized as total single-nucleotide substitution, but at nt 6991 and 14306, are characterized by single-nucleotide polymorphism.

The P47 stock was collected to extract total RNAs for NGS. The resultant data were processed and analyzed. **Figure 3A** exhibited a complete profile of sequencing depth cross the 16,536-nt-long antigenome (**Supplementary 1**), for which the average depth was 217×. The average depths of N, P, eGFP, M, F, H and L ORFs were determined to be 236×, 296×, 337×, 188×, 184×, 197× and 204×, respectively. The highest depth was 670×, separately at nt 1899 and 1900, across the antigenome. Two regions with the lowest depth, less than 30×, separately were 5’- and 3’-end sequences. The sequencing data were identified to have an approximately 99.9% of coverage range across the full-length antigenome (**Figure 3A** and **Supplementary 1**). Uncovered regions were located only at 5′- and 3′-end regions.

In general, the sequencing depth was positively correlated with the virus titer. The higher sequencing depth would generate the richer data of SNMs across a viral genome or antigenome. In our earlier study, the average sequencing depth for Senecavirus A was even more than 5,000×, due to a significantly high titer (10^9^ TCID_50_/mL) this virus could reach (Liu et al., 2021a). In contrast, the highest titer the rCDV-eGFP could reach was measured previously to be only 10^6^ TCID_50_/mL (Liu et al., 2021b). There thus was an extremely significant differentiation between their average sequencing depths. Even so, both the depth and the coverage were acceptable for the further analysis in the present study.

### A complete profile of mutations is uncovered

A total of nine SNMs were identified within the viral antigenome at P47, including five transversions (A1832C, G5022C, A5536C, T5580G and A14306C) and four transitions (G5746A, A6913G, A6991G and T8803C). **Figures 3B, C** showed absolute and relative sequencing depths for these nine SNMs, respectively. Out of them, SNMs at nt 1832, 5022, 5536, 5580, 5746, 6913 and 8803 were recognized as total single-nucleotide substitution, namely, 100% of mutation frequency, implying no remarkable quasispecies formation in the rCDV-eGFP population after repeated bottleneck passages. The other two SNMs, A6991G and A14306C, were characterized by single-nucleotide polymorphism (SNP). In the virological field, the SNP can be regarded as two or more different nucleotides coexisting at the same site in a viral population. Theoretically, successive plaque-to-plaque transfers can hardly lead to SNP arising in the viral genome. At nt 6991 and 14306, the original nucleotide was “A”, which underwent point mutations separately at these two sites, whereas both mutation frequencies were relatively low (**Figure 3C**). These two SNPs might be attributed to two extra rounds of common passaging at P46 and P47.

**Figure 4:**
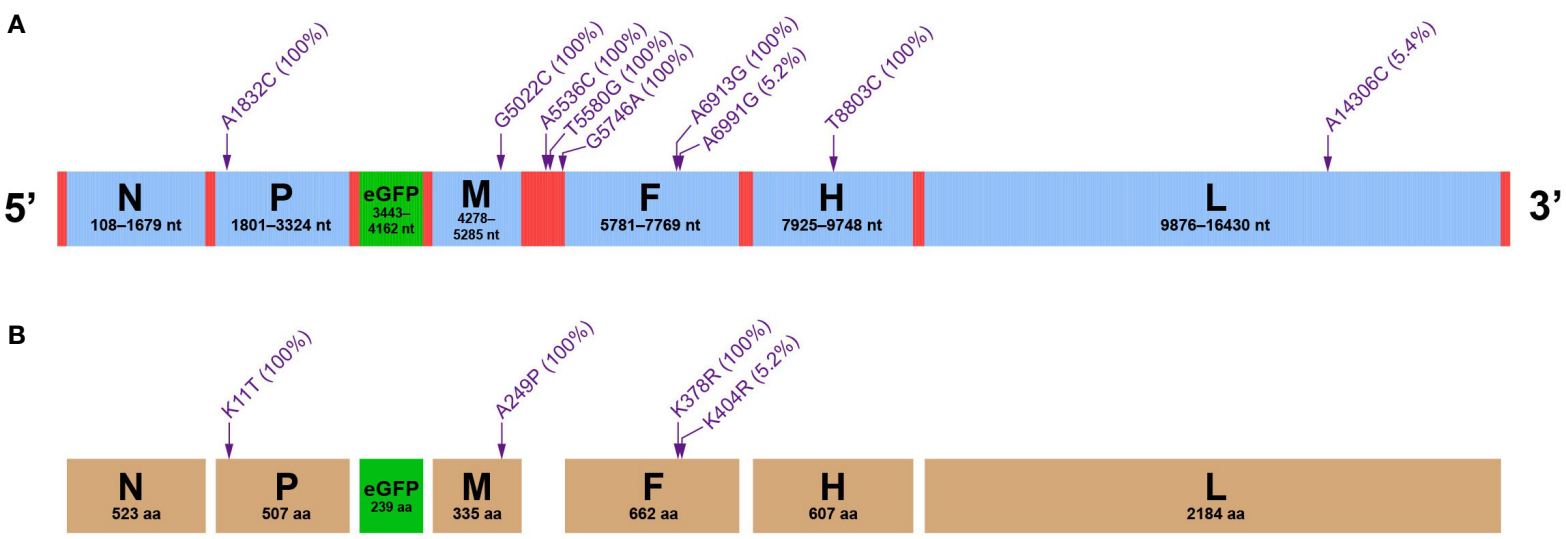
Mutation profiles of rCDV-eGFP at P47. Distribution of NGS-unveiled SNMs at rCDV-eGFP antigenome **(A)**. Distribution of SAAMs at structural proteins of rCDV-eGFP **(B)**. Arrow-indicated mutation sites do not exactly match their definite positions at antigenomes and at proteins. Mutation frequencies are enclosed within parentheses.

All nine sites of SNMs were schematically indicated in the full-length antigenome (**Figure 4A**). Mutation frequencies were enclosed within parentheses. Three SNMs, A5536C, T5580G and G5746A, were located at the untranslated region between M and F ORFs; the others were distributed in viral ORFs. Our previous study exhibited that two rCDV-eGFP stocks independently underwent serial common passages in ribavirin- and non-treated cell monolayers, consequently generating 62 and 23 SNMs in their individual antigenomes, respectively (Liu et al., 2021b). In comparison with our previous study, the present one indicated the pattern of plaque-to-plaque transfers leading to a low frequency of genomic mutation. Interestingly, none of the nine SNMs (**Figure 4A**) here was identified in our previous study (Liu et al., 2021b), suggesting that occurrence of SNM was a random event, regardless of the pattern of viral passaging.

The eGFP as a foreign protein is theoretically uninvolved in a series of viral events, *e.g.*, replication, transcription, regulation and packaging. Occurrence of SNM event hence was considered to be random, uncontrolled and retainable in the eGFP transcriptional unit during viral propagation (Liu et al., 2021c). For screening a progeny that bore SNMs as abundant as possible in the eGFP transcriptional unit, we once serially picked dimly fluorescent plaques out for the next round of plaque-to-plaque transfer. The NGS result, nonetheless, showed no SNM in the eGFP transcriptional unit. The mechanism remains to be elucidated.

Nine SNMs resulted in four single-amino acid mutations (SAAMs): P, M and F proteins revealed one (K11T), one (A249P) and two (K378R and K404R) SAAMs, respectively; the eGFP, N, H and L proteins had no SAAM (**Figure 4B**). Not all SAAMs have the same effect on function or structure of protein. The magnitude of this process depends on how similar or dissimilar mutated amino acids are, and on their own positions in proteins. SAAMs are generally classified into two types: conservative replacement and radical replacement (Hanada et al., 2007). Comparatively, the SAAM (A249P) at M protein was the radical replacement, and the other three were the conservative replacements, according to a uniform law for exchangeability of amino acids in proteins (Yampolsky and Stoltzfus, 2005). Three conservative replacements implied that these three SAAMs (K11T, K378R and K404R) might not affect the functions of P and F proteins.

### Variation of viral fitness remains to be elucidated

Plaque-to-plaque transfers of RNA viruses can cause accumulation of mutations and loss of fitness, as mentioned in subheading Introduction. However, this does not mean that repeated plaque-to-plaque transfers will disable viral propagation. Escarmís et al. (2002) subjected several low-fitness clones of foot-and-mouth disease virus (FMDV) to up to 130 plaque-to-plaque transfers, whereas no case of viral extinction could be documented (Escarmís et al., 2002). In the present study, we also found no case of viral extinction after serial plaque-to-plaque transfers, and rCDV-eGFP-induced plaques at P45 were still noticeable on cell monolayers. The passaging limit of rCDV-eGFP error catastrophe has not yet been determined.

The variation of viral fitness is perhaps neither continuous nor monotonic, but exhibiting a fluctuating pattern, for the period of serial plaque-to-plaque transfers. The probability of fitness values in bottleneck-passaged FMDV populations was proven to fit a Weibull distribution, rather than a log-normal distribution (Lázaro et al., 2003). FMDV, belonging to the family *Picornaviridae*, has a single-stranded, positive-sense RNA genome. In contrast, CDV has a single-stranded, but negative-sense RNA genome. Therefore, it is unclear whether the variation of rCDV-eGFP fitness also displays a fluctuating pattern during plaque-to-plaque transfers. As mentioned above, we found that a single dim plaque was able to induce brighter plaques on a cell monolayer in the next round of passaging, and *vice versa*. If dim and bright plaques are related to low and high viral fitnesses, respectively, the rCDV-eGFP fitness would vary in a fluctuating pattern during plaque-to-plaque transfers.

## Data availability statement

The datasets presented in this study can be found in online repositories. The names of the repository/repositories and accession number(s) can be found in NCBI PRJNA848641.

## Author contributions

JL and YJ performed experimental works. HZ and FZ completed data analysis. YZ, BN and FL provided funding. FL conducted experiments and wrote the manuscript. All authors contributed to the article and approved the submitted version.

## Funding

This work was supported by the Innovation Fund of China Animal Health and Epidemiology Center, and the Open Project Fund of State Key Laboratory of Microbial Technology, Shandong University (M2021-19).

## Acknowledgments

We gratefully thank Yu’s group in the Shanghai Tanpu Biotechnology Co., Ltd for performing NGS. We also thank other members in our group for their assistance in plaque-to-plaque passages.

## Conflict of interest

The authors declare that the research was conducted in the absence of any commercial or financial relationships that could be construed as a potential conflict of interest.

## Publisher’s note

All claims expressed in this article are solely those of the authors and do not necessarily represent those of their affiliated organizations, or those of the publisher, the editors and the reviewers. Any product that may be evaluated in this article, or claim that may be made by its manufacturer, is not guaranteed or endorsed by the publisher.
